# Semicarbazide-Sensitive Amine Oxidase Increases in Calcific Aortic Valve Stenosis and Contributes to Valvular Interstitial Cell Calcification

**DOI:** 10.1155/2020/5197376

**Published:** 2020-01-14

**Authors:** Nathalie Mercier, Sven-Christian Pawelzik, John Pirault, Miguel Carracedo, Oscar Persson, Bastien Wollensack, Anders Franco-Cereceda, Magnus Bäck

**Affiliations:** ^1^Inserm UMR_S 1116, Vandoeuvre-Lès-Nancy, France; ^2^Université de Lorraine, Vandoeuvre-Lès-Nancy, France; ^3^Translational Cardiology, Department of Medicine, Solna, Karolinska Institutet, Stockholm, Sweden; ^4^Theme Heart and Vessels, Division of Valvular and Coronary Disease, Karolinska University Hospital, Stockholm, Sweden; ^5^Department of Molecular Medicine and Surgery, Karolinska Institutet, Stockholm, Sweden

## Abstract

**Introduction:**

Calcific aortic valve stenosis (CAVS) is a common disease associated with aging. Oxidative stress participates in the valve calcification process in CAVS. Semicarbazide-sensitive amine oxidase (SSAO), also referred to as vascular adhesion protein 1 (VAP-1), transforms primary amines into aldehydes, generating hydrogen peroxide and ammonia. SSAO is expressed in calcified aortic valves, but its role in valve calcification has remained largely unexplored. The aims of this study were to characterize the expression and the activity of SSAO during aortic valve calcification and to establish the effects of SSAO inhibition on human valvular interstitial cell (VIC) calcification.

**Methods:**

Human aortic valves from *n* = 80 patients were used for mRNA extraction and expression analysis, Western blot, SSAO activity determination, immunohistochemistry, and the isolation of primary VIC cultures.

**Results:**

SSAO mRNA, protein, and activity were increased with increasing calcification within human aortic valves and localized in the vicinity of the calcified zones. The valvular SSAO upregulation was consistent after stratification of the subjects according to cardiovascular and CAVS risk factors associated with increased oxidative stress: body mass index, diabetes, and smoking. SSAO mRNA levels were significantly associated with poly(ADP-ribose) polymerase 1 (PARP1) in calcified tissue. Calcification of VIC was inhibited in the presence of the specific SSAO inhibitor LJP1586.

**Conclusion:**

The association of SSAO expression and activity with valvular calcification and oxidative stress as well as the decreased VIC calcification by SSAO inhibition points to SSAO as a possible marker and therapeutic target to be further explored in CAVS.

## 1. Introduction

Calcification of the aortic valve can evolve into calcific aortic valve stenosis (CAVS), which, if untreated, leads to heart failure. In the absence of a medical treatment, current options for severe symptomatic CAVS are limited to surgical or catheter-based prosthetic valve implantation. Oxidative stress could be one potential mechanism that increases valve calcification and CAVS disease burden [1, 2]. Valvular oxidative stress predominates around calcifying foci and enhances progression of CAVS. Acute H_2_O_2_-induced oxidative stress and the resulting higher reactive oxygen species (ROS) levels induce osteoblastic differentiation of human valvular interstitial cells (VIC), which are the main structural cells of the aortic valve [3]. These processes highly resemble those observed in atherosclerosis, in which, for example, vascular peroxidase 1, an enzyme generating H_2_O_2_, has been implicated in Ox-LDL-induced calcification of vascular smooth muscle cells [4].

Although the endogenous substrate is unknown, semicarbazide-sensitive amine oxidase (SSAO), also known as vascular adhesion protein-1 (VAP-1), is a mediator of tissue oxidative stress and a contributor to atherosclerotic plaque development [5]. The SSAO enzyme generates aldehydes, hydrogen peroxide (H_2_O_2_), and ammonia (NH_3_) from primary amines or amine groups within proteins. SSAO expression has been detected in human aortic valves, with significant upregulation in CAVS compared with noncalcified valves [6]. Furthermore, serum levels of SSAO are higher in patients with severe CAVS compared with patients presenting moderate CAVS and are significantly correlated with CAVS severity as assessed by echocardiography [7]. SSAO has been implicated in the differentiation of several cell types, such as chondrocytes and adipocytes [8–10], but its effects on VIC have not previously been investigated.

SSAO serves as a cardiovascular risk factor in particular for obese patients [11]. SSAO predicts an increased 9-year absolute risk of major cardiovascular events and cardiovascular mortality in subjects aged >50 years. Moreover, serum SSAO activity increases in types I and II diabetic patients compared with a nondiabetic control group [12]. Also, nicotine-enhanced oxidative stress through SSAO has been reported to contribute to the adverse effects of smoking [13]. Since obesity, diabetes, and smoking are also major risk factors for the incidence of CAVS [14–16], SSAO emerges as a common risk factor for atherosclerotic vascular disease and CAVS.

Taken together, the above observations converge to the hypothesis that oxidative stress through SSAO could be implicated in CAVS and could represent a novel target to develop anticalcification therapies. The aims of the present study were therefore (1) to determine the SSAO expression and activity in relation to the degree of calcification in human aortic valves, (2) to establish whether cardiovascular risk factors affect SSAO upregulation during valve calcification, and (3) to identify potential correlations between SSAO and other oxidative stress pathways. Finally, we aimed (4) to determine the role of SSAO in valve calcification by evaluating the potential of SSAO inhibition to inhibit human VIC calcification.

## 2. Material and Methods

### 2.1. Human Aortic Valves

Human aortic valves were obtained from 80 patients undergoing aortic valve replacement surgery at the Karolinska University Hospital in Stockholm, Sweden. The study was approved by the local ethics committee (2012/1633), and all patients gave informed consent.

### 2.2. Sample Collection and Macroscopic Dissection

Immediately after surgical removal, the valves were immersed in either RNA Later (Qiagen) and stored at 4°C until transport to the laboratory. For Western blot and SSAO activity determinations, valves were collected in phenol red-free DMEM supplemented with 10% fetal bovine serum (FBS), dissected, and stored at -80°C. Adjacent pieces were embedded in paraffin for histological analysis. Macroscopic dissection was performed dividing each valve into healthy, thickened, or calcified regions as previously described [17–19].

### 2.3. Valve mRNA Expression

Total RNA from aortic valves was isolated using the RNeasy Lipid Tissue Mini kit (Qiagen, Hilden, Germany). Quantification and the quality of RNA were assessed using a NanoDrop (Thermo Scientific, Waltham, MA, USA) and a 2100 Bioanalyzer (Agilent, Santa Clara, CA, USA), respectively. Valve gene expression data was obtained using Gene Chip Affymetrix human transcriptome 2.0 (HTA 2.0 arrays, Santa Clara, CA, USA) and normalized with a signal space transformation-robust multi-chip analysis (SST-RMA) using Expression Console (Affymetrix, Santa Clara, CA, USA) [18].

### 2.4. Western Blot and SSAO Activity Homogenates

Tissue specimens of human aortic valves were weighed and crushed in liquid nitrogen using a BioPulverizer (Biospec). The samples were suspended in 20 *μ*l of 1 mM NaH_2_PO_4_/Na_2_HPO_4_, pH 7.4 (NaPi buffer) containing 250 mM sucrose per mg of tissue. The tissue homogenates were centrifuged at 600x*g* for 5 minutes at 4°C, and the supernatants were stored at -80°C until use. The protein concentration was determined using DC Protein Assay (Bio-Rad, Bovine Gamma Globulin, S60) according to the manufacturer's protocol. Absorbance was read at 750 nm with FLUOstar® OPTIMA (BMG) 413-0149.

### 2.5. SSAO Activity

SSAO activity was analyzed by measurement of H_2_O_2_ production by a fluorimetric method adapted from Matsumoto et al. [20], using benzylamine as a substrate. Tissue homogenates (20 *μ*g of protein/well) were preincubated in duplicate in the absence or presence of the SSAO inhibitor semicarbazide (250 *μ*M, Sigma) for 15 minutes at 37°C in a total reaction volume of 50 *μ*l containing 40 mM NaPi buffer, 1 mM pargyline (Sigma), 1 IU/ml HRP, and 80 nM Amplex Red (Molecular Probes). Then, the SSAO substrate benzylamine (500 *μ*M, Sigma) was added. Incubation was carried out for 1 h at 37°C. H_2_O_2_ was used as the standard curve to determine the quantity generated in each condition. The fluorescence intensity was measured with a FLUOstar OPTIMA (413-0149) fluorescence microplate reader using excitation at 563 nm and fluorescence detection at 587 nm. To get the SSAO specific activity, the quantity of hydrogen peroxide detected with benzylamine in the presence of semicarbazide was subtracted from the hydrogen peroxide generated from the benzylamine in the absence of semicarbazide.

### 2.6. Western Blot

Tissue homogenates (10 *μ*g) were denatured in loading buffer (Laemmli Sample Buffer, BioRad) for 5 min at 95°C and loaded on an acrylamide SDS/PAGE gel (Bio-Rad) in a Mini-PROTEAN Tetra Cell (Bio-Rad). Resolved protein was subsequently transferred to a nitrocellulose membrane (Bio-Rad) with a Mini Trans-Blot Module. The membranes were blocked for 1 h at room temperature (RT) in 0.1% Tween 20 in TBS buffer (10 mM Tris-Base and 15 mM NaCl, pH 7.5) containing 5% nonfat dry milk and incubated with primary antibodies (rabbit anti-SSAO, clone H43, and anti *β*-actin, Santa Cruz Biotechnology, Dallas, TX, USA) in blocking solution. After several washes, the membrane was incubated with an appropriate horseradish peroxidase- (HRP-) coupled secondary antibody (anti-mouse or anti-rabbit IgG, GE Healthcare, Chicago, IL, USA) in blocking solution for 1 h at RT. Following washes, the protein bands were visualized using the Chemiluminescence Kit (Immun-Star™ WesternC™ Chemiluminescence Kit, Bio-Rad) on a Fujifilm Luminescent Image Analyzer LAS4000 system. The image analysis was performed with the Multi Gauge v3.0 program.

### 2.7. Histology and Immunohistochemistry

Aortic valve sections derived from 4 patients were deparaffinized in toluene, hydrated in ethanol at decreasing concentrations (100%, 95%, and 50%), and rinsed in distilled water before histochemical detection of calcification using alizarin red. For immunohistochemistry, deparaffinized sections were incubated in TBS-T 0.05%/3% H_2_O_2_ to block endogenous peroxidases. Nonspecific sites were blocked in TBS-T 0.05%/5% BSA (bovine serum albumin). The sections were then incubated with a primary anti-SSAO antibody (H-43, Santa Cruz Biotechnology, sc-28642) overnight at 4°C. After several washes in TBS-T 0.05%, sections were incubated with biotinylated donkey anti-rabbit secondary antibody (Jackson ImmunoResearch) for one hour at RT, followed by Streptavidin/HRP solution (Thermo Fisher Scientific, Waltham, MA, USA) for 10 min and DAB (3,3′-diaminobenzidine) (Vector Laboratories, SK-4100). Finally, some of the sections were counter-stained with haematoxylin (Vector Laboratories, H-3404) for one min and observed under an Eclipse Ci-S, 403116 microscope (Nikon).

### 2.8. VIC Isolation

Human aortic valves from *n* = 9 patients were digested for 16 h using an enzymatic cocktail containing collagenase I and dispase II as previously described [18]. Isolated VIC were seeded onto polystyrene tissue culture containers and cultured in DMEM supplemented with 10% FBS, 100 units/ml penicillin, 100 *μ*g/ml streptomycin, 1 mM sodium pyruvate, 10 mM HEPES, and 2 mM L-glutamine. Culture medium was changed every other day. Cells were used for experiments between passages 1 and 3. Cell culture reagents were purchased from Gibco, plastics were from Corning as previously described [18].

### 2.9. VIC Calcification

For measurement of *in vitro* calcification, VIC were seeded in a 96-well plate and incubated with standard medium or osteogenic medium containing 2.8 mM inorganic phosphate (Pi). Cells were treated with the specific SSAO inhibitor LJP1586 (1 *μ*M). Medium was changed every other day for eight to nine days. At the last medium change, 10 nM IRDye 800CW BoneTag Optical Probe (LI-COR, Bad Homburg, Germany) was added to all media, and VIC were incubated for another 24 h. Cells were washed three times with PBS, the plate was subsequently scanned on an Odyssey CLx near-infrared (NIR) fluorescence imager, and the obtained images were quantified using Image Studio Software Version 5.2 (LI-COR, Bad Homburg, Germany).

### 2.10. VIC TaqMan Real-Time PCR

Reverse transcription of mRNA isolated from VIC was performed using the High Capacity RNA-to-cDNA Kit (Thermo Fisher Scientific, Waltham, MA, USA) as previously described [21]. Quantitative real-time PCR was performed on a Quant7 Fast Real-Time PCR system (Thermo Fisher Scientific, Waltham, MA, USA) using TaqMan Assay-on-Demand Hs00907290_m1 (Thermo Fisher Scientific, Waltham, MA, USA) for SSAO. The relative mRNA expression of the target gene was quantified by the ΔCt method using TaqMan Assay-on-Demand Hs99999905_m1 for GAPDH as endogenous control.

### 2.11. Statistical Analysis

Results are expressed as mean ± SD. Statistical significance was assessed with one- or two-way ANOVA for repeated measurements followed by Holm-Sidak post hoc test for multiple comparisons. For comparisons of SSAO mRNA levels between different subgroups after stratification according to BMI, diabetes, and smoking, a two-way ANOVA was used taking into consideration the stratification groups and type of tissue (healthy, intermediate, and calcified tissue). Correlations were established using Spearman's correlations. Statistical significance was assigned at *P* < 0.05. To correct univariate analyses for multiple testing, we imposed a Bonferroni-corrected significance threshold of 0.007 (i.e., 0.05/7 univariate analyses). Statistical analyses were performed using SigmaPlot 12.5 (Systat Software Inc., USA).

## 3. Results

### 3.1. SSAO Expression, Activity, and Localization in Human Valves

**Figure 1:**
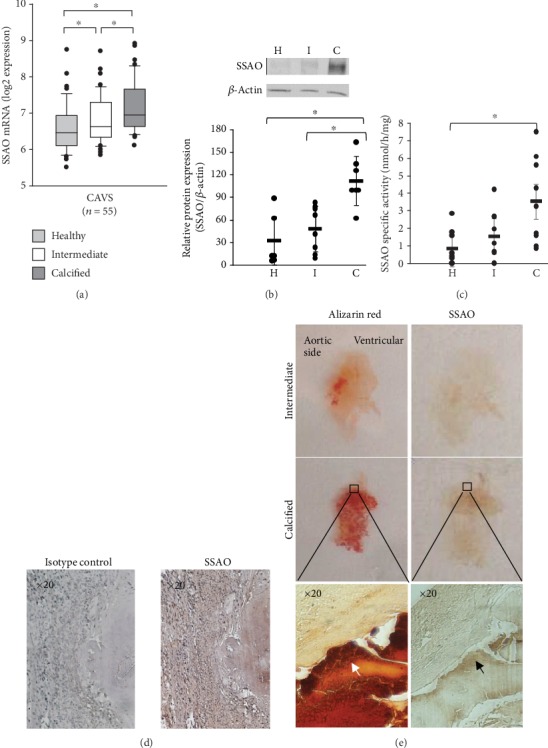
Expression and activity of SSAO are increased in calcified areas of human aortic valves. (a) SSAO mRNA expression in 55 human aortic valves derived from patients with CAVS was significantly increased in intermediate (I) and calcified (C) areas compared to healthy (H) valve tissue. (b) SSAO protein expression analysed by Western blot and quantified for *n* = 5 − 7 samples in each group. (c) Specific SSAO activity (*n* = 9) significantly increased in calcified (C) valve tissue. Data are presented as the mean ± SD. ^∗^*P* < 0.05 versus healthy valves. (d) Immunohistochemical stainings of SSAO in human aortic valves compared with isotype control (representative of *n* = 4). (e) Histological alizarin red (left panels) and SSAO immunohistochemical stainings (right panels) in an intermediate and in a highly calcified aortic valve showing high expression of SSAO in the proximity of calcified zones. The four upper panels present one entire cusp of an aortic valve and the lower panels show micrographs with a 20x magnification.

The levels of SSAO mRNA from human aortic valves derived from *n* = 55 patients with CAVS are shown in Figure 1(a). The SSAO mRNA expression was significantly and gradually upregulated in intermediately and fully calcified parts compared with healthy parts of the aortic valves (Figure 1(a)). SSAO protein levels measured in valves from *n* = 7 CAVS patients followed the same pattern of expression (Figure 1(b)). SSAO activity was significantly increased in calcified parts of *n* = 9 human aortic valves (Figure 1(c)). Immunohistochemical analysis revealed SSAO staining in valves derived from *n* = 4 CAVS patients (Figure 1(d)), with the most prominent signal in proximity to the alizarin red-stained calcified valves areas (Figure 1(e), left panels).

### 3.2. Valvular SSAO mRNA Expression in Relation to Obesity, Diabetes, and Smoking

**Figure 2:**
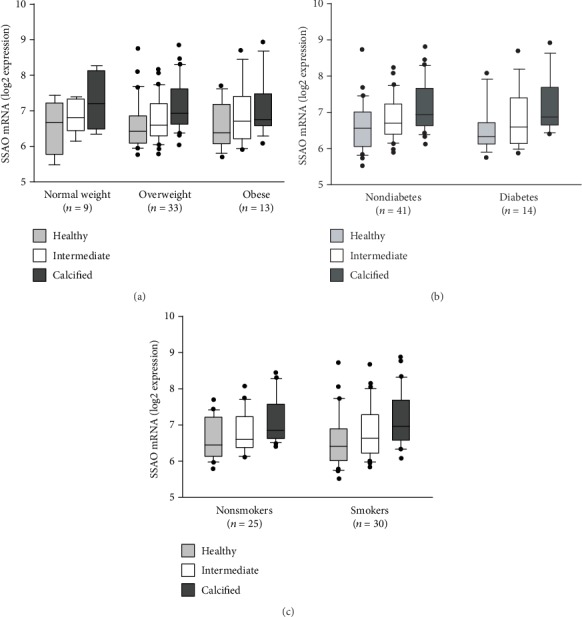
SSAO expression after stratification according to oxidative stress-associated risk factors for CAVS. SSAO mRNA expression levels in healthy, intermediate, and calcified aortic valve tissue. (a) Stratification according to body mass index (BMI): normal weight (BMI < 25), overweight (BMI: 25-30), and obese (BMI > 30). (b) Stratification according to the prevalence of type 2 diabetes mellitus. (c) Stratification according to smoking (nonsmokers versus current and former smokers). While the significant changes were retained for the type of tissue (healthy, intermediate, and calcified aortic valve tissue), no significant differences were detected between the different strata (two-way ANOVA).

Out of the *n* = 55 CAVS patients donating aortic valves for mRNA determinations, 9 patients were of normal weight (BMI: 18.5-25 kg/m^2^), 33 patients were overweight (BMI: 25-30 kg/m^2^), and 13 were obese (BMI: >30 kg/m^2^). Fourteen patients had type 2 diabetes and none had type 1 diabetes. There was one current smoker, 29 patients were former smokers, and 25 patients had never smoked. Stratification of patients based on BMI (Figure 2(a)), prevalent type 2 diabetes (Figure 2(b)), and smoking (Figure 2(c)) revealed that while the significant changes for the type of tissue (healthy, intermediate, and calcified tissue) were retained, no statistically significant differences in SSAO expression were detected between the different subgroups.

### 3.3. SSAO Expression in Relation to Pathways of Oxidative Stress

Correlation analysis of SSAO mRNA expression with other pathways associated with oxidative stress was performed in *n* = 64 human aortic valves with different degrees of calcification derived from patients with CAVS (*n* = 55) and aortic valve regurgitation (*n* = 9). Although the univariate analyses indicated significant correlations for SSAO with soluble serine hydroxymethyltransferase 1 (SHMT1) as well as with superoxide dismutases 2 and 3 (SOD2 and 3) in healthy tissue, none of these correlations were statistically significant after correction for multiple comparisons (Table 1, top row). However, correlations for SSAO with cytochrome b-245, alpha polypeptide (CYBA), and SOD3 achieved the Bonferroni-corrected statistical significance threshold (*P* < 0.007) in intermediate tissue (Table 1, middle row). Those correlations were, however, not statistically significant in calcified tissue. In contrast, SSAO significantly correlated with poly(ADP-ribose) polymerase 1 (PARP1) in calcified tissue (Table 1, bottom row).

### 3.4. Effect of SSAO Inhibition on VIC Calcification *In Vitro*

**Figure 3:**
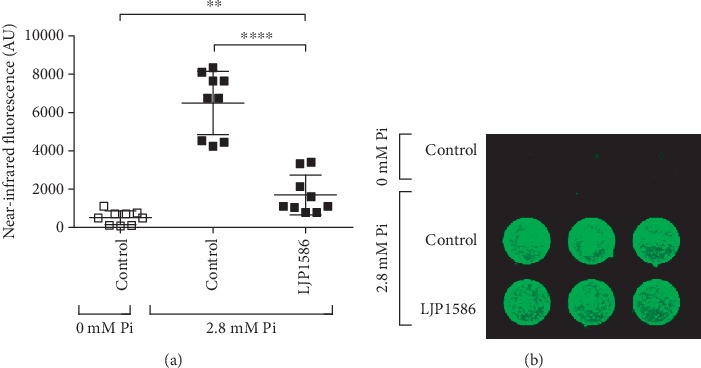
SSAO inhibition decreases calcification of human aortic valve interstitial cells. Valvular interstitial cells from aortic valves derived from *n* = 9 patients were cultured in the absence and presence of 2.8 mM inorganic phosphate (Pi). (a) The SSAO inhibitor LJP1586 (1 *μ*M) significantly inhibited calcification, measured as near-infrared fluorescence and quantified using Image Studio software. ^∗∗^*P* < 0.005; ^∗∗∗∗^*P* < 0.0001. (b) Valvular interstitial cells from one patient are exemplarily shown.

Primary cultures of VIC isolated from aortic valves from *n* = 6 CAVS patients expressed SSAO mRNA after 48 h of culture in the absence (ΔCt_control_ = 15.9 ± 1.45) and presence (ΔCt_osteogenic_ = 16.3 ± 1.34) of a high concentration of phosphate (2.8 mM) in the growth medium. Furthermore, SSAO activity was detected in VIC cultured under both conditions, and VIC SSAO activity was inhibited by 70% by the specific SSAO inhibitor LJP1586 (1 *μ*M). When VIC isolated from *n* = 9 CAVS patients were cultured under high phosphate conditions, calcification was significantly decreased by LJP1586 (1 *μ*M; Figure 3).

## 4. Discussion

This is the first report showing a gradual and significant increase in SSAO mRNA, protein, and activity in human aortic valves, which were divided into healthy, intermediate, and calcified tissue. The SSAO upregulation with valve calcification was independent of the cardiovascular and CAVS risk factors: obesity, diabetes, and smoking. Furthermore, a significant correlation of SSAO expression with pathways of oxidative stress was also revealed, with, in particular, a significant correlation with PARP1 in calcified tissue. Finally, we demonstrate that inhibition of SSAO activity decreased VIC calcification *in vitro*. Taken together, these results indicate a link between SSAO, oxidative stress, and aortic valve calcification and point to SSAO inhibition as a putative therapeutic approach to be explored for the prevention of valve calcification and CAVS progression.

A gradual upregulation of SSAO with the progression of aortic valve calcification is supported by the observational data on valvular mRNA expression in the present study. This was confirmed for protein expression by Western blot, which exhibited a similar pattern with significantly higher levels in calcified parts. Also, immunohistochemical analysis supported a localization of SSAO expression in proximity to calcified regions of the aortic valve tissue. The latter findings confirm previous results that have detected SSAO being upregulated in calcified stenotic aortic valves [6]. Moreover, our results extend the observation to show also increased SSAO activity in calcified valve tissue. We show that SSAO is present and functional in healthy areas of CAVS valves. The mean specific SSAO activity was approximately 800 pmol/h/mg, which is around 3 to 4 times higher compared with cartilage from human femoral knee joints [9]. Furthermore, we show that calcified parts of human aortic valves exhibited a 5.7 times higher SSAO activity compared to healthy parts of the same valves, supporting that SSAO mRNA and activity increase as the CAVS disease progresses. Indeed, a strong increase in SSAO expression and activity has also been described in other disease states, such as osteoarthritis human knee joints [9].

When expressed and active in a tissue, SSAO generates H_2_O_2_ and hence contributes to the local oxidative stress. SSAO is also increased in several disease states associated with an increased systemic oxidative stress. Interestingly, obesity [11, 14, 22], diabetes [12, 15], and smoking [16, 23] are all examples of cardiovascular risk factors associated with both increased SSAO expression and an increased risk of incident CAVS. In the present study, we did, however, not detect any significant changes in the pattern of valvular SSAO mRNA expression after stratification according to BMI, prevalent diabetes, or smoking status, suggesting that the observed upregulation of SSAO mRNA with calcification was not affected by systemic states of increased oxidative stress.

SSAO is also related to other pathways of oxidative stress [24–26]. In the present study, we show that the correlations of SSAO with oxidative stress pathways vary at different disease stages. Whereas no significant correlations were revealed in healthy valve tissue, we identify CYBA and SOD3 mRNA levels as being significantly correlated with SSAO mRNA in intermediate stages of valve calcification. CYBA, also known as p22phox, is an essential component of superoxide generating NADPH oxidase (Nox) complex and is increased in osteoblasts around calcifying foci [27]. The lack of correlation for SSAO with Nox4 in the present study supports a differential regulation of Nox subunits in calcification processes [27]. Surprisingly, SSAO was significantly associated with SOD3 in intermediate but not calcified valve tissue, which may represent a defense mechanism. SOD3 and catalase are downregulated with aortic valve calcification and in CAVS [28]. Indeed, SOD3 and catalase adenoviral transfection to VIC reestablishes an impaired DNA damage response and reduces early markers of VIC activation [28]. SOD3 dismutation hence requires concomitant catalase to degrade H_2_O_2_ for a full antioxidative effect. Inhibiting additional H_2_O_2_ generation by SSAO may potentially serve to limit oxidative stress in CAVS. Importantly, SSAO exhibited a significant correlation only with PARP1 in calcified valve tissue. PARP1 is a nuclear enzyme activated by DNA strand breakage and oxidative stress. Interestingly, PARP1 correlates with disease severity in CAVS and is induced by proinflammatory stimulation of human VIC [29]. PARP1 may directly participate in soft tissue calcification by means of releasing extracellular poly(ADP-ribose) in response to oxidative and/or DNA damage [30].

The addition of H_2_O_2_ in the Pi-induced calcification model further increased calcium deposition *in vitro*, and VIC derived from calcified valves were more susceptible to oxidative stress compared with VIC from healthy valves [3]. Oxidative stress also induces the expression of Runx2, a transcriptional factor of osteoblasts [27], expressed by VIC under osteogenic conditions [31]. To determine the mechanisms behind the observed associations of SSAO expression with valve calcification, we finally show SSAO mRNA expression and activity in VIC and that inhibition of SSAO by LJP1586 decreased calcification of VIC *in vitro*. These findings are in line with studies of SSAO expression in chondrocytes, in which the hypertrophic differentiation is delayed by LJP1586, a response that is accompanied by lower expression of calcification markers such as alkaline phosphatase, osteopontin, and MMP9. These findings suggest that inhibiting SSAO in valves may slow down the mineralization process.

Some limitations of the present study must be acknowledged. The observational data on SSAO expression in human aortic valves cannot determine the causal relation of the findings. We can also not rule out that correlation of SSAO with PARP1 and other markers of oxidative stress are not dependent on unknown covariates. Finally, we cannot exclude that the expression of SSAO by other cell types in the aortic valve, such as endothelial cells and dendritic cells [32], may contribute to disease progression.

## 5. Conclusion

This work provides evidence that, in addition to being a possible disease marker, SSAO could be implicated in the mechanism of valve calcification. This could offer a new therapeutic perspective for CAVS.

## Figures and Tables

**Table 1:** Spearman correlations (Rho) for SSAO mRNA with mRNA levels of poly(ADP-ribose) polymerase 1 (PARP1), soluble serine hydroxymethyltransferase 1 (SHMT1), NADPH oxidase 4 (NOX4), cytochrome b-245, alpha polypeptide (CYBA), and superoxide dismutases (SOD) 1-3 in healthy, intermediate, and calcified tissue from human aortic valves. Bonferroni-corrected significance threshold is 0.007.

Healthy	**PARP1**	**SHMT1**	**NOX4**	**CYBA**	**SOD1**	**SOD2**	**SOD3**
*Rho*	0.045	0.32	-0.059	0.052	-0.11	0.28	0.33
*P*	0.722	0.011	0.642	0.682	0.399	0.027	0.0075
*N*	64	64	64	64	64	64	64

Intermediate	**PARP1**	**SHMT1**	**NOX4**	**CYBA**	**SOD1**	**SOD2**	**SOD3**
*Rho*	0.26	0.27	0.20	0.41	0.042	0.26	0.34
*P*	0.036	0.031	0.12	0.00096	0.74	0.043	0.0064
*N*	64	64	64	64	64	64	64

Calcified	**PARP1**	**SHMT1**	**NOX4**	**CYBA**	**SOD1**	**SOD2**	**SOD3**
*Rho*	0.37	-0.17	-0.075	0.21	-0.21	0.055	-0.11
*P*	0.0031	0.18	0.56	0.099	0.093	0.67	0.38
*N*	64	64	64	64	64	64	64

## Data Availability

The data used to support the findings of this study are included within the article.
